# A Comparative and Evaluative Study of Two Cytological Grading Systems in Breast Carcinoma with Histological Grading: An Important Prognostic Factor

**DOI:** 10.1155/2014/767215

**Published:** 2014-12-08

**Authors:** Pinki Pandey, Alok Dixit, Subrat Chandra, Swarn Kaur

**Affiliations:** ^1^Department of Pathology, U. P. Rural Institute of Medical Sciences & Research, Saifai, Etawah 206301, India; ^2^Department of Pharmacology, U. P. Rural Institute of Medical Sciences & Research, Saifai, Etawah 206301, India; ^3^Department of Pathology, RML Institute of Medical Sciences, Lucknow, India; ^4^Department of Pathology, BPS Medical College, Khanpur, Sonepat, Haryana, India

## Abstract

*Objective*. Cytonuclear gradings in the breast carcinoma raise the level of FNA reportage and improves patient management. Our aim was to evaluate and compare two cytological grading methods (Robinson's and Mouriquand's) in breast carcinoma and correlate it with Nottingham modification of Scarff-Bloom-Richardson (SBR) histological grading. *Materials and Methods*. 30 cytologically proven cases of infiltrating ductal carcinoma were graded cytologically and histologically. Cytograding was done by Robinson's and Mouriquand's methods (grades I to III) followed by comparison of the two methods. Cytogradings were correlated with SBR grading method. Sensitivity, specificity, diagnostic accuracy, and concordance and discordance rates were evaluated. *Results*. An overall concordance of 76.66% between cytogradings, of 83.33% between Robinson's method and SBR, and of 66.66% between Mouriquand's method and SBR was seen. Robinson's method correlated best with SBR in all the three nuclear grades. Robinson's method showed a diagnostic accuracy of 90% with 91.30% sensitivity while Mouriquand's method had an accuracy of 76.66% with 95.65% sensitivity. The specificity by Mouriquand's method was quite low (14.28%) as compared to Robinson's method (85.71%). *Conclusion*. Comprehensive cytological grading of breast cancer by Robinson's method seems better because of more objective set of criteria, easy reproducibility, and specificity.

## 1. Introduction

Breast lesions account for one of the largest groups of conditions necessitating pathological, radiological, and surgical intervention. Breast cancer is one of the leading causes of cancer death in women. It is emerging as the leading cause of cancer mortality in Indian women, with nearly 80,000 new cases of breast cancer being diagnosed annually in India [1]. In spite of the advances in cancer research its annual incidence is increasing by 1% [2]. It is of relevance to get a proper diagnosis in such lesions to alleviate the anxiety of the patient and to start treatment at the earliest possible moment.

The value of histological typing and grading of breast carcinomas is well established and has gained a strong foothold. As neoadjuvant therapy is becoming increasingly common for the treatment of early breast cancer, it is desirable to grade the tumor preoperatively on FNAC so that the most appropriate medical regimen could be selected. The advantages of FNAC in diagnosing breast cancer have been known a long time ago but its utility in grading the breast cancer has been largely underestimated and very few studies [3–7] have been conducted on cytological nuclear grading. Hence, cytological grading may assume prime importance for the patient, who may benefit from specific treatment modalities (e.g., tamoxifen and herceptin) prior to the resection of the tumors and in those who present with metastases. Thus, cytoprognostic grading will provide valuable information to the treating oncologist to plan the management. The present study has been carried out with the aim of evaluating and comparing the two cytological nuclear grading methods on fine needle aspirates of breast carcinoma and comparing cytological and histological grading systems.

## 2. Materials and Methods

This was a prospective study conducted in the department of pathology in our institution. 30 cytologically proven cases of ductal carcinoma breast and their corresponding histopathology were included in the study. Papanicolaou stained FNA smears and haematoxylin and eosin (H&E) stained tissue sections were evaluated for cytological and histological grading, respectively. A prior approval was obtained from Institutional Ethics Committee for conducting the study.

Cytological grading was done using the grading systems described by Robinson et al. [8] and Mouriquand and Pasquier [9]. The Robinson grading system takes into account six cytological parameters (Table 1). A value between one and three was given to every factor analyzed. Scores for each of the six cytologic features were added together to give a total score for each case. In each case the final score ranged between 6 and 18.

Mouriquand's grading system takes into account the following criteria: cellular characters (clustering, 0/isolated cells, 3), nuclear features (anisokaryosis, 2; large size, 3; budding, 2; naked, 3; hyperchromasia, 2; hypochromasia, 3), nucleoli (blue, 2; red, 3), and number of mitoses (≥3 per slide = 1, ≥6 per slide = 3). Based on these criteria, breast cancers were graded into grade I (score ≤ 5), grade II (score 5–9), and grade III (score ≥ 10).

Histological grading was performed on formalin-fixed paraffin embedded sections from mastectomy specimens using Nottingham modification of Scarff-Bloom-Richardson system [10] using 0.44 mm diameter of microscopic field. Three parameters were taken into consideration: degree of tubule formation, nuclear pleomorphism, and number of mitoses. Each parameter was scored between one and three. Thus the overall score for all cases ranged between three and nine.

*Statistical Analysis*. A comparison between cytological gradings obtained by two methods and the histological grade was done and sensitivity, specificity, diagnostic accuracy, and concordance and discordance rates were calculated.

## 3. Results

All the 30 cases of carcinoma breast were from female patients. Age of patients ranged between 40 and 49 years. Right sided breast was predominantly involved (63.33%). In most of the cases, the lump was found in the upper outer quadrant (50%) and the average size was 1–4 cm. Mean duration of breast carcinoma was 3 months ranging from 3 to 12 months. In 60% of cases, the lump was freely mobile while in the rest was fixed. Nipple retraction was present in 30% of cases, Peau d'orange appearance in 20% of cases, and ulceration of the skin in 10% of cases.

Using aspirate samples, the Robinson method showed more cases (26.66%) of grade I compared to Mouriquand's method, which showed only 6.66% cases. A higher proportion (83.33%) of tumors was categorized as grade II by Mouriquand's method as compared to Robinson's method (60%). Proportion of grade III cases by both methods was almost similar (13.33% by Robinson's method and 10% by Mouriquand's method).

Of the 8 grade I cases by Robinson's method, 2 were graded as grade I, whereas 6 cases were overgraded as grade II by Mouriquand's method. Of the 4 grade III cases by Robinson's method, 3 cases were graded as grade III by Mouriquand's method, and one case was undergraded as grade II. Discordant cases were seen in the group of grade II tumors by Mouriquand's method. Out of 25 grade II tumors by Mouriquand's method, only 18 (60%) were grade II by Robinson's method. A complete concordance in grading by both methods was obtained in 23 (76.66%) of the 30 patients (Table 2).

**Figure 1:**
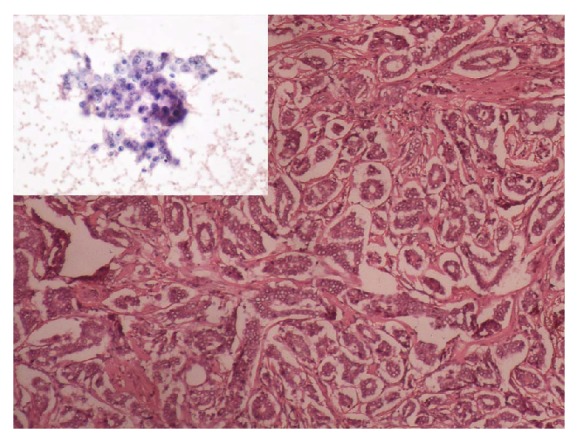
Infiltrating ductal carcinoma histological grade I—predominantly tubular differentiation, mild variability in nuclear size and shape, and uniformly dispersed nuclear chromatin. Inset (Robinson's cytological grade I): loosely cohesive cluster of monomorphic ductal epithelial cells exhibiting mild nucleomegaly (1-2 times the size of erythrocytes), smooth nuclear margins with small nucleoli (H&E, ×100; inset: PAP, ×400).

**Figure 2:**
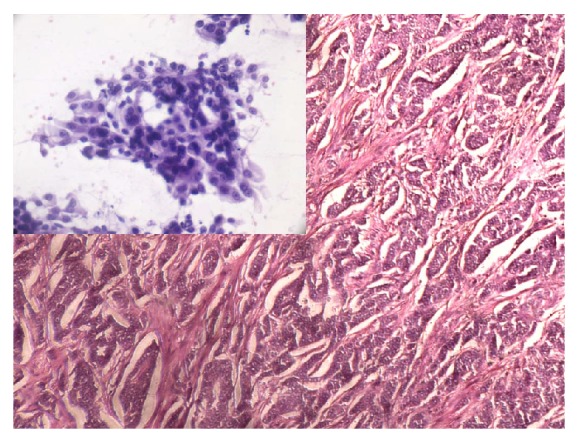
Infiltrating ductal carcinoma histological grade II—exhibiting cords and islands with tubular differentiation, mild variation in nuclear size and shape, vesicular nucleus, and conspicuous nucleoli. Inset—less cohesive cluster of tumor cells with moderate pleomorphism, moderate nucleomegaly (3-4 times the size of erythrocytes), slight irregularity in nuclear envelope, and conspicuous nucleoli (Robinson's cytological grade II) (H&E, ×100; inset: PAP, ×400).

**Figure 3:**
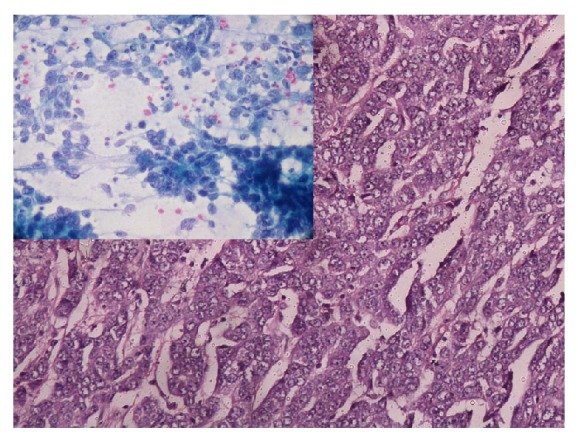
Infiltrating ductal carcinoma histological grade III—very minimal tubular differentiation, marked nuclear pleomorphism, coarse nuclear chromatin, prominent nucleoli, and brisk mitosis. Inset—highly pleomorphic tumor cells with marked nucleomegaly (>5 times the size of erythrocytes), irregular nuclear membrane, prominent nucleoli, and coarsely clumped chromatin (Robinson's cytological grade III) (H&E, ×200; inset: PAP, ×400).

Table 3 illustrates the correlation of Nottingham modification of Scarff-Bloom-Richardson grading system on histopathology with the two cytological nuclear grading methods. Out of 7 cases that had been judged histopathologically to be grade I, 6 cases (85.74%) were found to be grade I by Robinson's method (Figure 1) contrasting only 14.28% cases by Mouriquand's method. Best correlation was observed in grade II tumors; 84.21% cases of the Robinson's method (Figure 2) and 89.47% cases of Mouriquand's method were correlated with histopathology by Nottingham modification of Scarff-Bloom-Richardson grading system. Among the grade III tumors, the cytohistological correlation was seen in 75% and 50% cases by Robinson's (Figure 3) and Mouriquand's methods, respectively.

**Figure 4:**
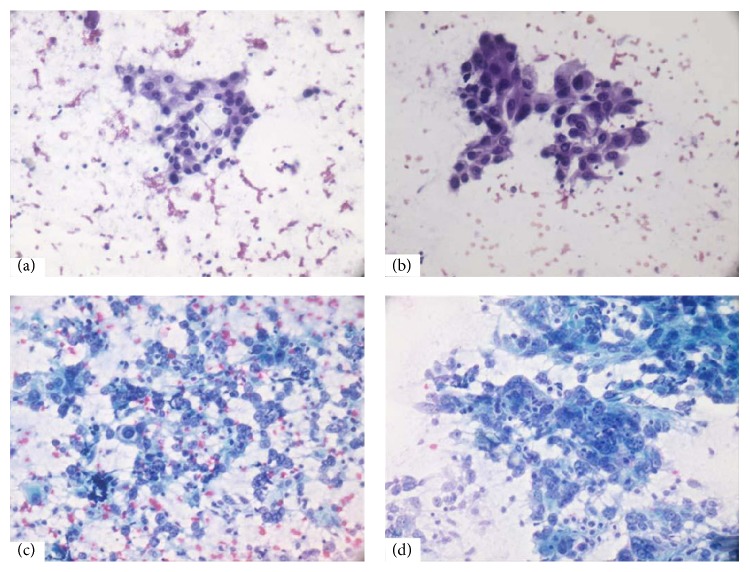
(a) Loosely cohesive cluster of mild pleomorphic ductal epithelial cells with mild nucleomegaly, smooth nuclear envelope, and indistinct nucleoli—Robinson's cytological grade I (PAP, ×400). (b) Moderately pleomorphic ductal cells with slightly irregular nuclear membrane and noticeable nucleoli. Nuclei are 3-4 times the size of erythrocytes—Robinson's cytological grade II (PAP, ×400). (c and d) Robinson's cytological grade III—markedly pleomorphic discohesive singly scattered tumor cells with marked nucleomegaly, irregular nuclear membrane, prominent abnormal nucleoli, and coarsely clumped nuclear chromatin ((c) PAP, ×400; (d) PAP, ×400).

The concordance rates between the two cytological grades were initially assessed and further compared with the histological grade. There was an overall concordance of 83.33% between the Robinson method and histological grading system, whereas 66.66% concordance was seen between Mouriquand's method and Nottingham modification of Scarff-Bloom-Richardson grading method as shown in Table 4. On further breakup into different grades, maximum concordance was seen only in grade II lesions by Mouriquand's method, whereas Robinson's method correlated best with those of Nottingham modification of Scarff-Bloom-Richardson grading method in all the three nuclear grades (Figure 4) (85.74% in grade I, 84.21% in grade II, and 75% in grade III lesions) (Table 3).

In order to statistically evaluate which of the two cytological grading methods corresponded better to the histological grading, grade I cases were considered as low grade and both grades II and III cases were clubbed together as high grade in both cytological and histological grading systems. Each cytological grading system was separately compared with the histological grading system. Table 4 illustrates the comparison of statistical parameters and concordance of the two cytogradings with histological grading system.

## 4. Discussion

The evaluation of pathological prognostic factors in preoperative breast samples is a worthwhile activity. Being able to predict the biological behavior of a cancerous breast lesion on cytology would have considerable advantages, as it would help select the most appropriate treatment. Cytoprognostic grading identifies fast growing tumors (grade III) which are more likely to respond to chemotherapy than low grade slow growing tumors which may be better suited to pretreatment with tamoxifen. This also allows assessment of tumor in situ, so that not only the most suitable treatment could be selected before primary surgery but also the morbidity associated with overtreatment of low grade tumors could be avoided [8].

Nuclear grading was first introduced by Black and Speer [11] and refined by various workers till a composite cytonuclear grading system was introduced by Robinson et al. [8] which has been used in this study.

In our study we have assessed nuclear grade in FNAC smears and compared it with nuclear grade in the respective surgical specimens. Similar to the studies by previous workers [3–5, 8], grade II tumors comprised the predominant group, both by Mouriquand's and Robinson's cytological grading systems (83.33% and 60%, resp.).

Not many studies directly comparing the two grading methods could be found in the literature. Only one study conducted by Das et al. [3] was retrieved from the literature. Similar to the study by Das et al. [3], the concordance between the two cytograding methods was found to be 76.66%; however, Wani et al. [5] showed a high degree of concordance (90.9%). Some degree of discordance between the two cytograding methods was observed in all the grades with the majority of the discordant cases observed in grade II tumors which is in agreement with the study by Das et al. [3]. The reason appears to be the presence of mitosis in most of the discordant cases. Mitosis is one of the parameters used for grading by Mouriquand's method, whereas mitosis is not taken into consideration in Robinson's method.

Similar to the studies by previous workers [12, 13], the overall concordance of Robinson's grading with histological grading was 83.33% which was higher than comparison of Mouriquand's grading with histological grading of 66.66%. Das et al. [3] graded 52 breast carcinomas by Robinson's and Mouriquand's cytological grading systems and found that they have similar concordance (71.2%) with histological grading. In the study by Sinha et al. [6] the overall concordance was 69.5%. Pandit and Parekh [14] graded 75 breast carcinomas by Robinson's method and showed 64% concordance with Nottingham modification of Scarff-Bloom-Richardson grading system on histopathology. Among the three cytonuclear grading methods (Robinson's, Fischer's modification of Black's, and Scarff-Bloom-Richardson's) studied by Bhargava et al. [7], Robinson's grading system was found to have the best correlation with histological grades as well as ER/PR expression. Ohri et al. [15] found an agreement of 95% between simplified Black cytological grade and histological grade.

In our study the rate of discordance of Robinson's grading with histological grading was 16.66%, which was in contrast to the study by Robinson et al. [16] and Das et al. [3] where the rate of discordance was relatively higher (39.5% and 28.8%, resp.). This was accounted for by tumor heterogeneity and observer subjectivity when assessing nuclear grade. The number of discordant cases of Mouriquand's grading with histological grading (33.33%) in the present study was comparable to other studies [14, 17]. These findings suggested that Robinson's method of cytological grading was a reasonably reliable method of grading breast carcinoma in FNAC smears. Nuclear grading on cytological specimens has been shown to correlate well with that of histological sections unlike other parameters like tubule formation and mitotic count. Tubule formation was difficult to assess in FNAC smears though some authors [8, 18] believed that cell clustering or dissociation reflected tubule formation. This might be responsible for the discordance observed between the cytological and histological grading systems.

In the present study, Robinson's method showed the diagnostic accuracy of 90% whereas 76.66% was established by Mouriquand's method. The sensitivity (91.30% and 95.65%) of both Robinson's and Mouriquand's methods was similar. However, the specificity by Mouriquand's method was quite low (14.28%) as compared to Robinson's method (85.71%). Meena et al. [19] compared 100 cases of breast carcinoma and found that the sensitivity and specificity of cytological grading system were 90.77% and 84.42%, respectively.

In our study, although the number of cases was fewer (30), we found that Robinson's cytological grading was used more accurately than Mouriquand's method since it emphasizes nuclear features which are highlighted to greater extent in Papanicolaou and Giemsa stained smears. As far as cytohistological correlation is concerned, Robinson's cytological grading correlated better than Mouriquand's cytological grading with histological grading system. This is because Robinson's grading method has two more criteria that are cell dissociation and uniformity which is not present in Mouriquand's cytological grading system. Also Robinson's cytological grading was more specific than Mouriquand's method, when Nottingham modification of Scarff-Bloom-Richardson grading system was considered as gold standard. The criteria for grading a tumor by the Robinson cytological method were simpler and easier to reproduce as compared to the Mouriquand method. Kalhan et al. [20] have shown that nuclear morphometry can be applied to augment the cytological grading of breast cancer. They also suggested that nuclear perimeter, when coupled with clinicopathological features, may be used to prognosticate and classify the patients into low-risk and high-risk groups.

A seemingly constant criticism of tumor grading is that it is a subjective evaluation and therefore inherently lacks reproducibility. Thus, clinicians often place little reliance on this valuable prognostic parameter and a potentially useful consultation with a pathologist by the clinician does not occur. It is, however, important to draw the reader's attention to the fact that a best comprehensive cytological grading of the breast cancers is possible by the method proposed by Robinson because of well-defined set of criteria, better reproducibility, and specificity. Despite the limitation of small sample size, our study indicates that adoption of cytonuclear grading system in breast carcinoma aspirates goes far to raise the level of FNA reportage as well as improves overall patient management.

## 5. Conclusion

As neoadjuvant therapy is becoming increasingly common for the treatment of early breast cancer, it is desirable to grade the tumor preoperatively on FNAC so that the most appropriate medical regimen could be selected. FNAC also helps in evaluating the aggressiveness of tumor and can be used as prognostic marker for better management of patient. Robinson's grading system for breast cancer provides more objective set of criteria, easy reproducibility, and specificity as compared to Mouriquand's system of grading.

## Figures and Tables

**Table 1:** Cytological grading with the method of Robinson et al. [8].

Criterion	Scores		
	1	2	3
Cell dissociation	Mostly clusters	Single cells, clusters	Mostly single cells
Nuclear size	1-2 times size of RBCs	3-4 times	More than 5
Cell uniformity	Monomorphic	Mildly pleomorphic	Pleomorphic
Nucleoli	Indistinct/small	Noticeable	Abnormal
Nuclear margin	Smooth	Slightly irregular/folds	Buds, clefts
Chromatin pattern	Vesicular	Granular	Clumping/clearing

Grade I: scores 6–11; Grade II: scores 12–14; Grade III: scores 15–18.

**Table 2:** Comparison of cytological grading by Robinson's and Mouriquand's methods.

Robinson's grading		Mouriquand's grading			Concordance
		I	II	III	
I	8 (26.66)	2	6	—	23/30 (76.66)
II	18 (60)	—	18	—	
III	4 (13.33)	—	1	3	
Total	**30**	** 2 (6.66)**	** 25 (83.33)**	** 3 (10)**	

Figures in parenthesis indicate percentage.

**Table 3:** Correlation of Nottingham modification of Scarff-Bloom-Richardson method on histopathology with two cytological nuclear grading methods.

Nuclear grading on histopathology	Number of cases	Nuclear grading on cytology					
		Robinson's method			Mouriquand's method		
		I	II	III	I	II	III
I	7	6 (85.74)	1	—	1 (14.28)	6	—
II	19	2	16 (84.21)	1	1	17 (89.47)	1
III	4	—	1	3 (75)	—	2	2 (50)

Figures in parenthesis indicate percentage.

**Table 4:** Comparison of statistical parameters and concordance of the two cytological grading methods with histological grading method.

Statistical parameter	Robinson's cytological grade	Mouriquand's cytological grade
Sensitivity	91.30%	95.65%
Specificity	85.71%	14.28%
Diagnostic accuracy	90.0%	76.66%
Number of concordant cases with histological grade	25/30 (83.33%)	20/30 (66.66%)
Number of discordant cases with histological grade	5/30 (16.66%)	10/30 (33.33%)
